# Navigation and Self-Semantic Location of Drones in Indoor Environments by Combining the Visual Bug Algorithm and Entropy-Based Vision

**DOI:** 10.3389/fnbot.2017.00046

**Published:** 2017-08-29

**Authors:** Darío Maravall, Javier de Lope, Juan P. Fuentes

**Affiliations:** Department of Artificial Intelligence, Faculty of Computer Science, Universidad Politécnica de Madrid Madrid, Spain

**Keywords:** visual bug algorithm, entropy search, visual topological maps, internal models, unmanned aerial vehicles

## Abstract

We introduce a hybrid algorithm for the self-semantic location and autonomous navigation of robots using entropy-based vision and visual topological maps. In visual topological maps the visual landmarks are considered as leave points for guiding the robot to reach a target point (robot homing) in indoor environments. These visual landmarks are defined from images of relevant objects or characteristic scenes in the environment. The entropy of an image is directly related to the presence of a unique object or the presence of several different objects inside it: the lower the entropy the higher the probability of containing a single object inside it and, conversely, the higher the entropy the higher the probability of containing several objects inside it. Consequently, we propose the use of the entropy of images captured by the robot not only for the landmark searching and detection but also for obstacle avoidance. If the detected object corresponds to a landmark, the robot uses the suggestions stored in the visual topological map to reach the next landmark or to finish the mission. Otherwise, the robot considers the object as an obstacle and starts a collision avoidance maneuver. In order to validate the proposal we have defined an experimental framework in which the visual bug algorithm is used by an Unmanned Aerial Vehicle (UAV) in typical indoor navigation tasks.

## Introduction

Some of the most challenging behaviors of autonomous robots are related to navigation tasks. According to a policy of safe navigation, the basic level of behaviors are devoted by strategies that allow the obstacle detection and the collision avoidance. Once these tasks have been conveniently solved, the next level is route planning, i.e., the generation of routes or paths that allow the robot to reach specific places in the environment. Some information about the environment is needed for such kind of planning. This information is managed by the robot control system and concerns the fundamental issue of environment mapping. The robot can store a sort of metric data related to the environment, topological information, including relationships between elements of the environment and features, maybe visual, associated to them, or any combination of both.

The aim of our work is to introduce an efficient method for autonomous robot navigation supported by visual topological maps (Maravall et al., 2013a), in which the coordinates of both the robot and the goal are not needed. More specifically, this novel method is meant to drive the robot toward a target landmark using self-semantic location, while simultaneously avoiding any existing obstacle using exclusively vision capacity. By self-semantic location we refer to the concept used generally in topological models that differs from metric navigation models. Therefore, self-semantic location means that the robot control system is able to determine an approximate, semantic self-location when a landmark already presented in the model is detected and recognized. In summary, self-semantic location refers to the cognitive information associated to a particular place of the environment (e.g., “I am at the restaurant,” so that the robot has a specific cognitive framework at this particular place of the environment).

The proposed algorithm is based on a conventional bug algorithm, although in our version we use only visual information as opposite to the classic versions that employ metric information. As it is well-known the bug algorithms are a family of techniques for obstacle avoidance in robot navigation with metric maps in real-time (Lumelsky and Stepanov, 1987; Lumelsky, 2005). These techniques make the robot head toward the goal and, if an obstacle is encountered, it circumnavigates it and remember how close it gets to the goal. Once the obstacle is avoided, the robot returns to the closest point and continues toward the goal. The main drawback of the conventional metric bug algorithms is that they need the knowledge of the robot localization (the hardest constraint) besides the coordinates of the goal in a common reference framework. To update the robot's position coordinates is necessary the use of external positioning systems.

Our method focuses on the search of the target visual landmark based on the entropy maximization of the images captured by the robot (Fuentes et al., 2014), which is used when the robot is in an unknown localization. Our hypothesis is that there is a direct and positive correlation between the entropy of an image and the probability of this image containing one or several objects: the higher the entropy, the higher its probability of containing several objects inside it; and conversely, the lower the entropy, the higher its probability of containing a single object inside it. Afterwards, when the robot has detected a potential landmark, i.e., it is in a known location, a dual architecture is executed with inspiration on cerebellar system of living beings combined with a brain activity. This dual architecture provides the reactive and anticipatory behaviors for the robot autonomous control.

Although the technique can be applied to any type of autonomous robot, we employ an Unmanned Aerial Vehicle (UAV). The application of our algorithm to these flying robots is justified due to occasionally the UAVs only have available an onboard camera as main input sensor for obstacle detection and landmark and location recognition as well as other specific sensors for flying related matters. This is mainly the case with Micro Aerial Vehicles (MAVs).

The rest of the paper is organized as follows. The next section summarizes some related work with the main findings in this work, namely navigation based on topological maps and obstacle detection and avoidance. After the theoretical foundations of the entropy-based search combined with the bug algorithm, we present the experimental work performed for its validation using an UAV. The paper ends with some suggestions for future research work based on the use of the vehicle onboard cameras for vision-based quality inspection and defects detection in different operating environments.

## Related work

Our proposal involves the use of visual graphs, in which each node stores images associated to landmarks, and the arcs represent the paths that the UAV must follow to reach the next node. Therefore, these graphs can be used to generate the best path for an UAV to reach a specific destination, as it has been suggested in other works. Practically each traditional method used in ground robots for trajectory planning has been considered for aerial ones (Goerzen et al., 2010). Some of those methods use graph-like models and generally they use algorithms such as improved versions of the classic A^*^ (MacAllister et al., 2013; Zhan et al., 2014) and Rapidly-exploring Random Tree Star (RRT) (Noreen et al., 2016) or reinforcement learning (RL) (Sharma and Taylor, 2012) for planning. RL is even used by methods that consider the path-planning task in cooperative multi-vehicle systems (Wang and Phillips, 2014), in which coordinated maneuvers are required (Lopez-Guede and Graña, 2015).

On the other hand, the obstacle avoidance task is also addressed here, which is particularly important in the UAV domain because of a collision in flight surely implies a danger and the partial or total destruction of the vehicle. Thus, the Collision Avoidance System (CAS) (Albaker and Rahim, 2009; Pham et al., 2015) is a fundamental part of control systems. Its goal is to allow UAVs to operate safely within the non-segregated civil and military airspace on a routinely basis. Basically, the CAS must detect and predict traffic conflicts in order to perform an avoidance maneuver to avoid a possible collision. Specific approaches are usually defined for outdoor or indoor vehicles. Predefined collision avoidance based on sets of rules and protocols are mainly used outdoors (Bilimoria et al., 1996) although classic methods such as artificial potential fields are also employed (Gudmundsson, 2016). These and other conventional well-known techniques in wheeled and legged robots are also considered for being used in UAVs (Bhavesh, 2015).

Since most of the current UAVs have monocular onboard cameras as main source of information several computer vision techniques are used. A combination of the Canny edge detector and the Hough transform is used to identify corridors and staircases for trajectory planning (Bills et al., 2011). Also, feature points detectors such as SURF (Aguilar et al., 2017) and SIFT (Al-Kaff et al., 2017) are used to analyze the images and to determine free collision trajectories. However, the most usual technique is optic flow (Zufferey and Floreano, 2006; Zufferey et al., 2006; Beyeler et al., 2007; Green and Oh, 2008; Sagar and Visser, 2014; Bhavesh, 2015; Simpson and Sabo, 2016). Sometimes optic flow is combined with artificial neural networks (Oh et al., 2004) or other computer vision techniques (Soundararaj et al., 2009). Some of those techniques (de Croon, 2012) are based on the analysis of the image textures for estimating the possible number of objects that are captured. Finally, as in other scopes of research, deep learning techniques are also been used to explore alternatives to the traditional approaches (Yang et al., 2017).

## The visual bug algorithm

In words the visual bug algorithm can be summarized as follows: “search the target landmark and once it is visible then move always toward the target landmark and circumnavigate any existing obstacle when necessary.”

Besides the implementation of the visual search procedure (which is obviously less critical when the robot controller is provided with the orientation ⊖ to the target landmark), the critical element is the robot odometry (Lumelsky and Stepanov, 1986) and more specifically the robot's dead reckoning or path integration, for this reason the robot planner is then based in the control of the orientation ⊖ toward the target landmark using topological maps.

**Figure 1:**
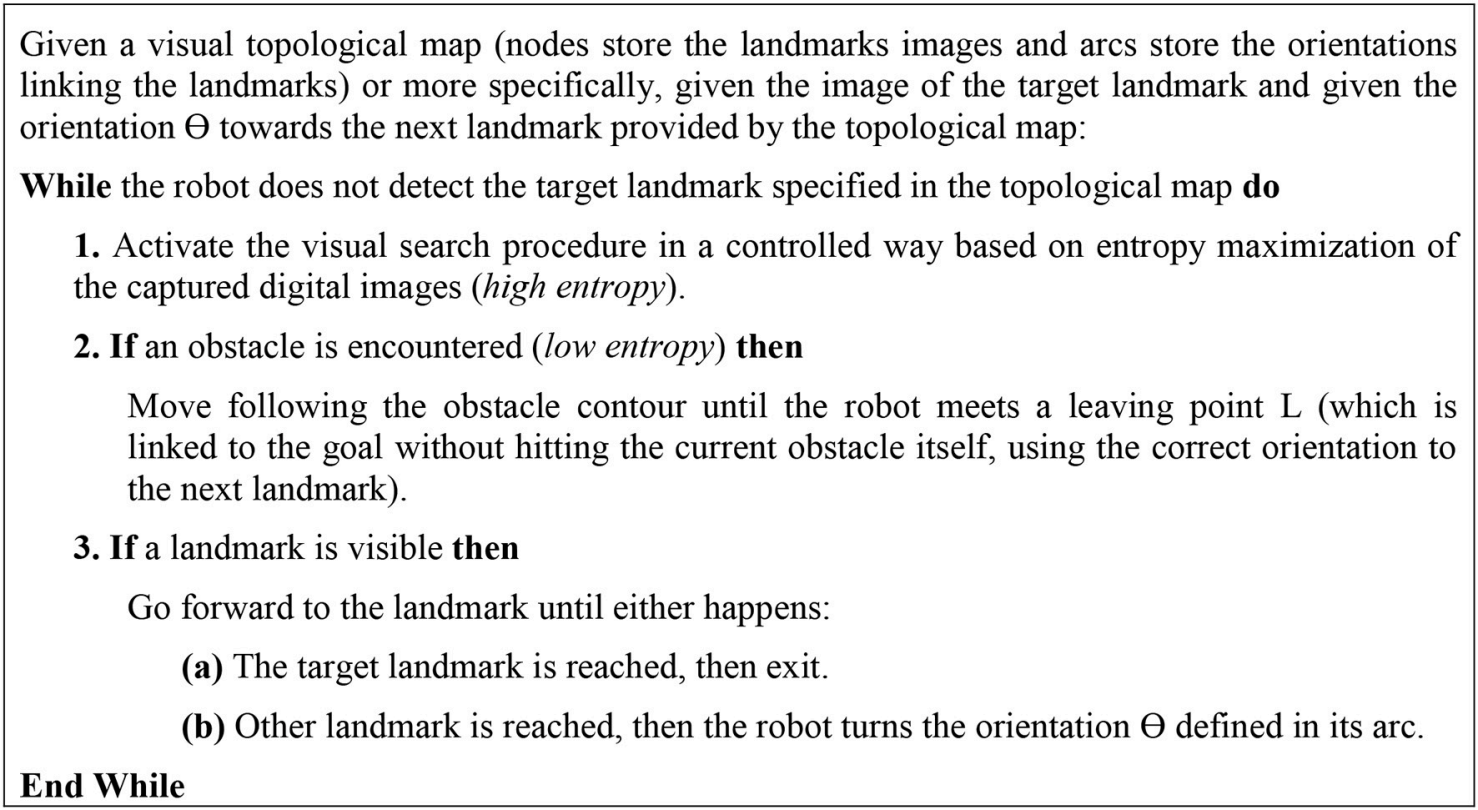
Visual Bug Algorithm.

Figure 1 shows the pseudocode of the visual bug algorithm (Maravall et al., 2015a):

Besides the basic procedure visual search, this algorithm consists of three additional complex vision-based robot behaviors:

- B1: “go forward to the goal”
- B2: “circumnavigate an obstacle”
- B3: “turns the orientation ⊖ ”

There are defined three basic environment's states or situations:

- S1: “the goal is visible”
- S2: “there is an obstacle in front of the robot”
- S3: “a landmark from the topological map is visible”

The visual bug algorithm sets out also hard computer vision problems for both the perception of the environment's states S_1_, S_2_, and S_3_ and for the implementation of the three basic robot's behaviors B_1_, B_2_, and B_3_, so that it is devoted the bulk of the remaining sections of the paper to describe our proposals aimed at solving these specific computer vision problems.

## Visual landmarks search and recognition for robot self-semantic location using visual topological maps

**Figure 2:**
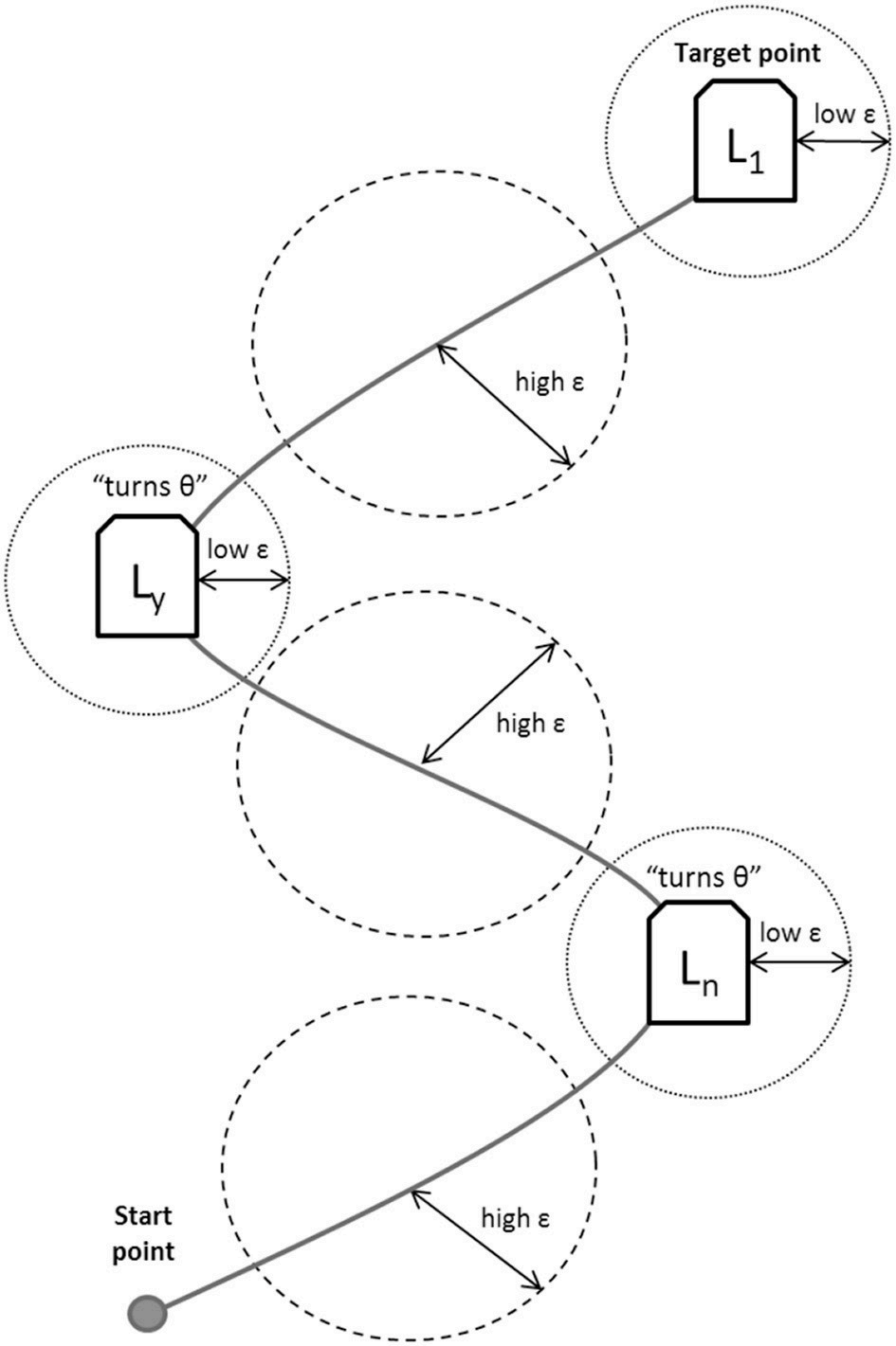
A Visual Topological Map is a graph defined as a set of nodes (visual landmarks) with relations based on arcs (orientations).

As it was already pointed out in the pseudocode of the visual bug algorithm, and as it is shown in Figure 2, the visual topological map is a connected graph where each node stores images corresponding to the next nodes/landmarks to which it is connected, and toward which the robot must navigate. Besides, the arcs connecting two nodes stores information about the relative orientation, Θ, of one node/landmark with respect to the other. Hence, this hybrid visual and “odometric information” (just orientations and landmarks) can be used by the robot's navigation module for its self-semantic location, allowing the search and detection of the sequential nodes/landmarks belonging to a specific route.

Notice to the existence of unknown states (high error ε) in which the robot uses entropic vision; known states (low error ε) are used when the robot is able to determinate its position with respect to the L_n_ landmark.

**Figure 3:**
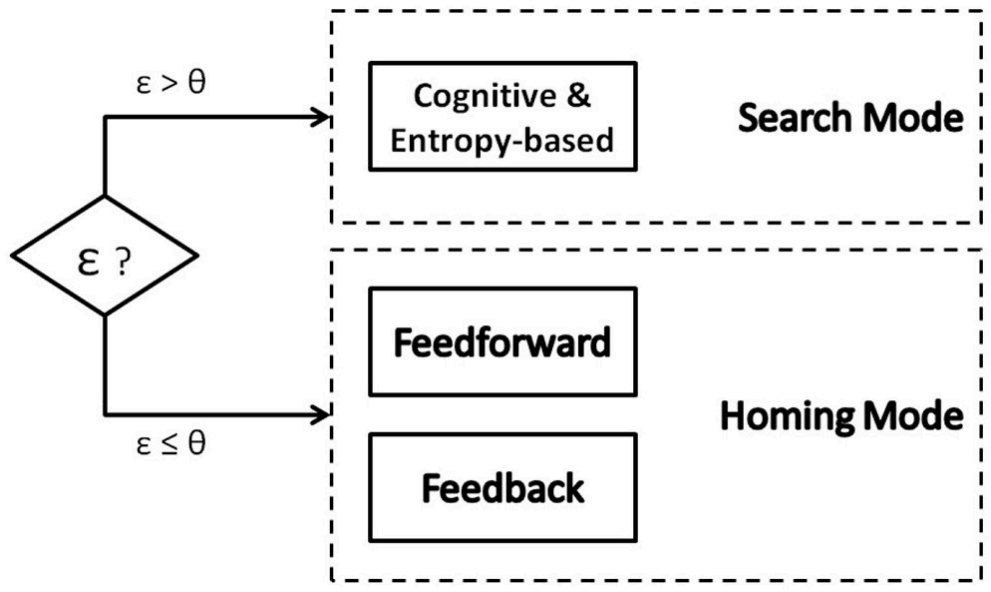
Finite state automaton for Search or Homing Mode selection.

In the sequel a novel method for this self-semantic location task is proposed (i.e., the task of landmark search and recognition; Maravall et al., 2013b) based on the combination of image entropy for landmark search (*Search Mode*), and a dual feedforward/feedback vision-based control loop (*Homing Mode*) for the final landmark homing. Figure 3 shows the finite state automaton that models the robot's controller between the *Search Mode* [S] and the *Homing Mode* [H] depending on the magnitude of the error ε or the difference images (respect to the landmarks defined in the topological map) and according to the simple heuristic rule: If {error is big} Then {S} Else {H}. As previously mentioned, once the image containing the candidates for the target landmark has been obtained by the entropy maximization process in the *Search Mode*, the robot's controller switches to the *Homing Mode* to guide the UAV toward the target landmark (Maravall et al., 2015a).

### Search mode: entropy-based landmark search

**Figure 4:**
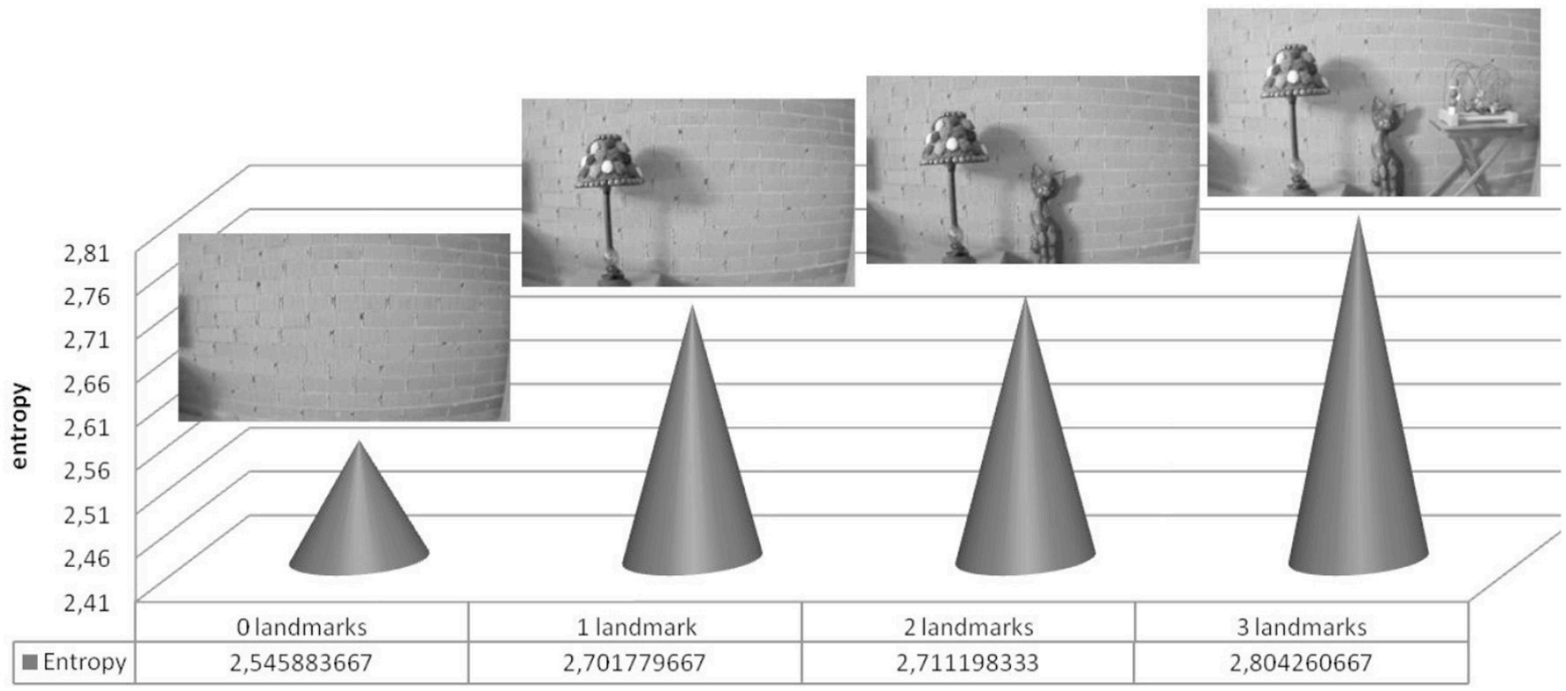
Example of the empirical fact that the higher the number of objects (landmarks) inside an image, the higher its entropy.

The main idea behind the entropy-based search is the direct and positive correlation between the entropy of an image and the probability of the image of containing several objects inside (in the case of high entropy) or conversely the probability of the image of containing just a single object (in the case of low entropy) (Fuentes et al., 2014). Figure 4 shows an example of this idea.

As visual landmarks in topological maps are usually selected as outstanding single objects, normally surrounded by other objects that produce together complex images with a high entropy, the task of visual landmarks search and detection can be formalized as a sequential process of image entropy maximization aimed at converging to an image of high entropy, hopefully containing several candidates of the single target object landmark, followed by a homing process aimed at guiding the robot toward the target landmark by means of a vision-based loop control, as explained below.

Therefore if it is represented by *u* the robot's control variables (angles of movement as *pitch, roll* and *yaw*) this process of entropy maximization can be expressed as follows:

(1) $$\begin{array}{r} \dot{u}=+\frac{\partial \text{H}\left[\text{Hist}\left({\text{I}}_{\text{k}}\right)\right]}{\partial u} \end{array}$$

where H is the image entropy, as given by the standard definition (Shannon, 1948) of the entropy of the normalized histogram Hist(I_*k*_):

(2) $$\begin{array}{r} \text{H}\left[\text{Hist}\left({\text{I}}_{\text{k}}\right)\right]=\;-\sum \left[Hist\left(I_{k}\right)\cdot \log_{2}Hist\left(I_{k}\right)\right] \end{array}$$

These control signals are obtained along the *k* trials, during the operation of the robot in the flight environment.

### Homing mode: the feedforward/feedback controller for landmark homing

This homing mode has been implemented as a dual feedforward/feedback control architecture which is constituted by the combination of a feedback module (either based on a conventional PD control or on error gradient control), and a feedforward module (based on either a neurocontroller or a memory-based controller).

**Figure 5:**
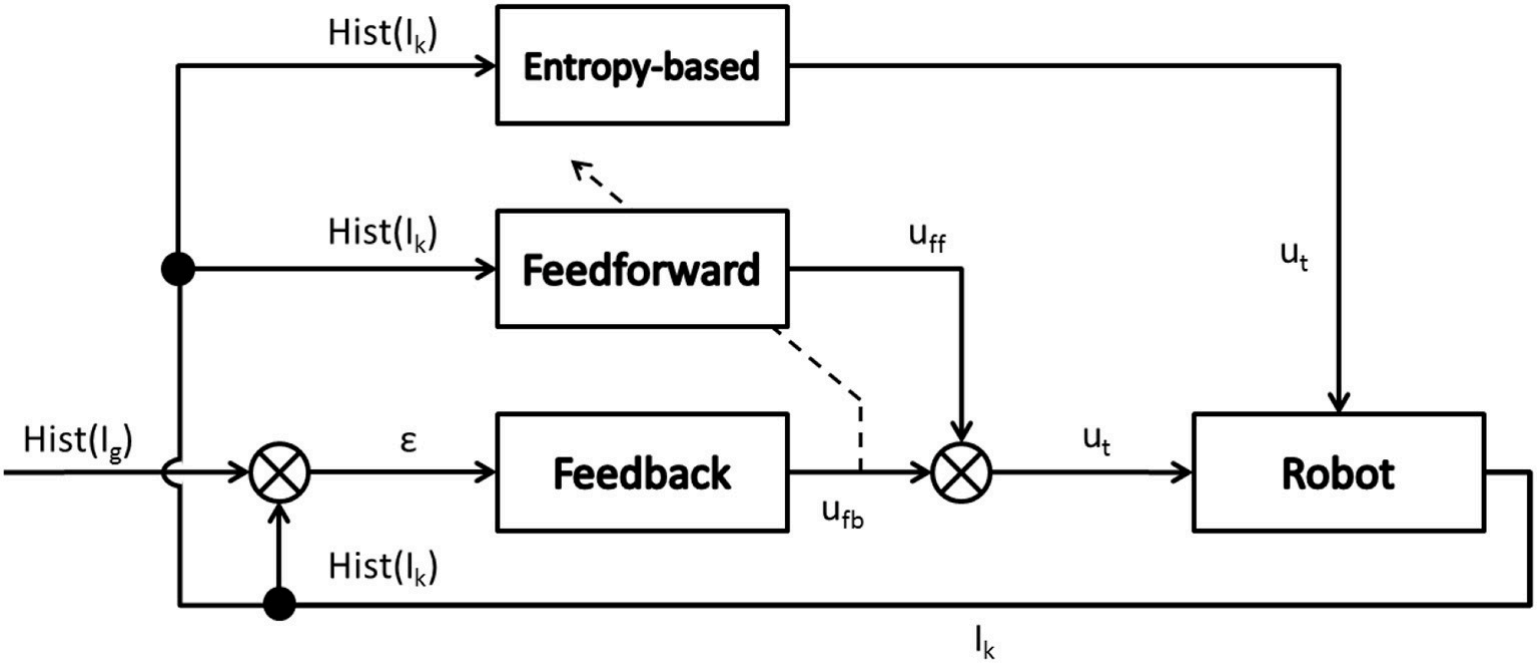
The feedforward/feedback controller.

This dual control architecture is shown in Figure 5, which shows the block-diagram of the dual feedforward/feedback controller (Kawato, 1990). Notice that the feedback or reactive controller receives as input the ε error; this vision-based error signal ε is obtained as the difference between the histogram of the recognized landmark or histogram of the goal image Hist(I_g_) and the histogram of the current image Hist(I_k_) during the *k* iteration of the controller.

Nevertheless, the feedforward or anticipatory controller receives as input the histogram of the current image Hist(I_k_). The feedback controller is implemented as a conventional PD control algorithm (Maravall et al., 2013a) whose control parameters are set experimentally. On the other hand, the feedforward controller is based on an inverse model (Kawato, 1999) using a conventional neural network based on multilayer perceptron (Maravall et al., 2015b), which is trained (using the output of the feedback controller) when error signal ε has been reduced in the last iterations. Both output control signals u_fb_ and u_ff_ are combined as follows:

(3) $$\begin{array}{r} {\text{u}}_{\text{t}}\;={\text{w}}_{\text{fb}}\;\cdot {\text{u}}_{\text{fb}}\;+{\text{w}}_{\text{ff}}\;\cdot {\text{u}}_{\text{ff}} \end{array}$$

The weight parameters w_fb_ and w_ff_ are set experimentally along the trials with the UAV. The u_*t*_ is the vector control signals (*pitch, roll*, and *yaw*) which is sent to the robot.

Summarizing, when the error ε is high, the robot is around an unknown state and it executes the entropy-based controller as it has been detailed in the previous section. When the robot recognizes the current location and it is able to detect any landmark already stored in the topological map, it follows the commands generated by the dual feedforward/feedback controller.

## Experimental work: UAV navigation based on the visual bug algorithm plus landmarks search through image entropy

### AR.Drone

**Figure 6:**
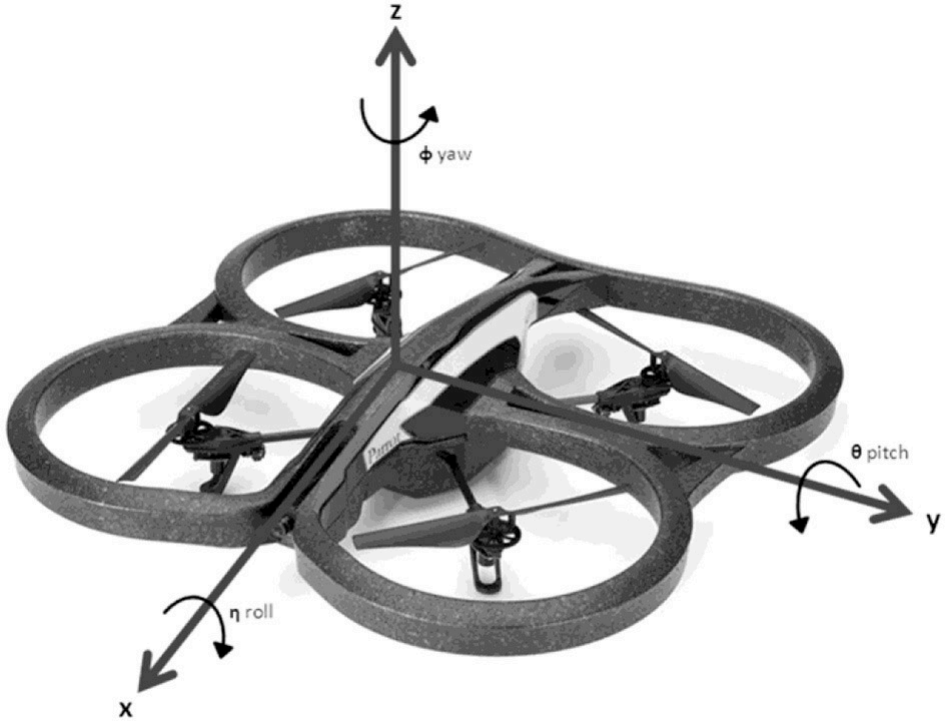
AR.Drone 2.0 with axes: roll, pitch, and yaw.

For the experimental work concerning the testing and validation of the proposed method for visual landmarks search and detection, it has been used the quadrotor Parrot AR.Drone 2.0 (Figure 6) as UAV well-established and widely available robotics research platform (Krajnik et al., 2011).

The Parrot company launched the project named AR.Drone with the final objective of producing a micro UAV aimed at both the mass market of videos games and the home entertainment. AR.Drone 2.0 has been finally released on the market and it is widely available at a low price that make it possible to be used as a unique robotic platform for experimental work on UAVs.

All commands and images can be exchanged with a central controller via an *ad-hoc* Wifi connection. The AR.Drone has an on-board HD camera and it has four motors to fly through the environment. This UAV supports four different control signals or degrees of freedom along the usual axes (*roll, pitch, gaz*, and *yaw*). The different maneuvers can be executed by the axes grades (*roll, pitch, gaz*, and *yaw*). The *gaz* variable regulates the altitude control.

This UAV has been extensively used for autonomous navigation at indoor environments (Maravall et al., 2013a).

### Description of the experiments

**Figure 7:**
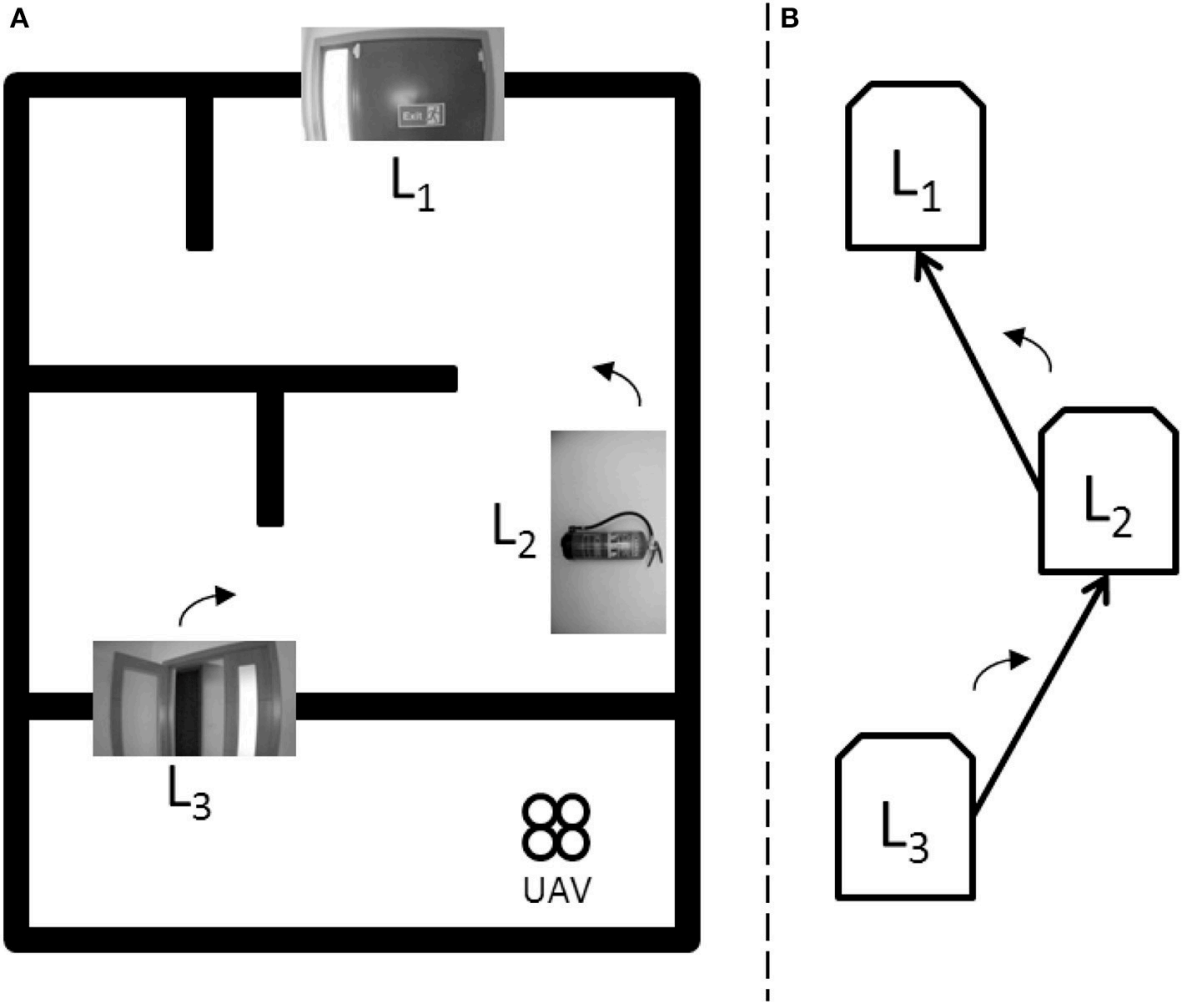
Schematic information about the experimental environment. **(A)** Experimental indoor environment schema. **(B)** Associated visual topological map.

For the validation of the proposed algorithm, it has been selected a typical indoor environment (see Figure 7). In an emergency situation, an UAV can help people to find an exit door along a secure route. For this, a topological map has been defined for the representing the environment, and to verify how the UAV is able to search and reach an exit door when an emergency situation occurs (for example fire in a building, flood in a home, etc.).

Figure 7 shows the environment used during the experiments with the UAV, for which it is defined a visual topological map using a set of landmarks situated along the environment: (a) the experimental indoor environment schema and (b) its associated visual topological map. The UAV navigates along the environment using the entropy-based controller, and when a landmark (a node) is detected through the dual controller, the UAV performs the control signals stored (in the arc), to guide it to the next landmark defined in the map.

For this scenario, the topological map defines three landmarks L_n_: the landmark L_3_ is an open door, the landmark L_2_ is a fire extinguisher and the landmark L_1_ or landmark goal is an exit door. Figure 7 also shows the approximate situation of each landmark along the environment.

### Discussion of the experimental results

The UAV is located on a given start point (unknown state) in the experimental indoor environment, at a given distance near with respect to a specified landmark in the topological map. The UAV aims reach this first landmark using the entropy-based controller because the error ε is high.

Through the entropy-based controller, it is executed a process of image entropy maximization (Search Mode) aimed at converging to a state of high entropy, hopefully containing landmarks from the visual topological map. This controller uses three entropic values: the left zone (H_L_), the right zone (H_R_), and the central zone (H_C_) of image captured from the UAV.

When the UAV is near of the first landmark L_3_, the error ε decreases, and it is switched to the Homing Mode, through the dual feedforward/feedback controller. Therefore, the dual controller starts generating control signals u_t_ increasingly optimal, for send them to the UAV.

The signals u_fb_ have been calculated by the feedback controller (reactive behavior) from the different images that are captured by the UAV's onboard camera, as well as the signals u_ff_ provided by the feedforward controller (anticipatory behavior). Both signals have been consolidated adaptively through interaction of the robot with its environment.

**Figure 8:**
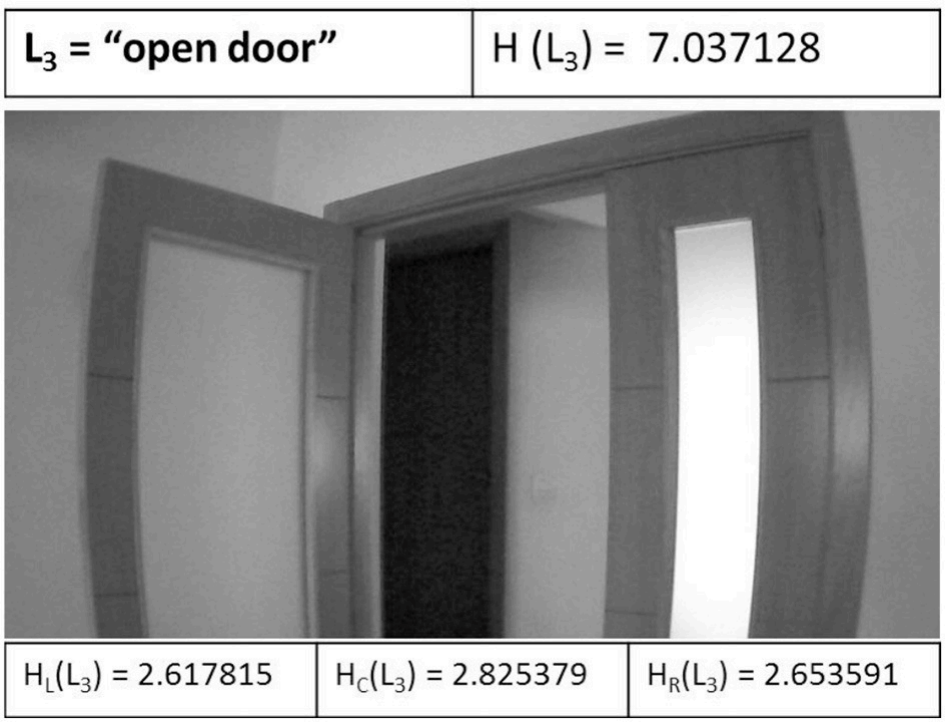
The first landmark L_3_ when is detected by the UAV.

Figure 8 shows the first landmark L_3_ when is detected by the UAV. The entropic values are shown: the full entropy of image H(L_3_), the left entropy H_L_(L_3_), the center entropy H_C_(L_3_), and the right entropy H_R_(L_3_) of the image captured. H_C_(L_3_) is the higher entropy, therefore the UAV executes a maneuver go forward to the open door.

The UAV recognizes the L_3_ landmark at the *k*=*4* iteration. When the UAV reaches the specified landmark, it will proceed to execute the specified maneuver in the corresponding arc in the topological map. It is considered that the approximation maneuver has been executed correctly and the UAV has been able to approximate to this landmark for its identification. The arc stores the corresponding orientation Θ, for which the UAV performs a forward maneuver (*pitch*) plus a right turn (*yaw*), passing this way the open door that has been identified. The attitude control (*gaz*) remains constant throughout the experiment (about 150 cm above the ground).

At this point the UAV is in an unknown state again and the entropy-based controller is activated. After several iterations, the robot locates a state with higher entropy in the environment and executes an approximation maneuver to the fire extinguisher. When the error ε decreases, it is switched to the dual controller and the L_2_ landmark is detected at the *k* = *11* iteration. The UAV performs a left turn (*yaw*) to the next landmark defined in the visual topological map.

**Figure 9:**
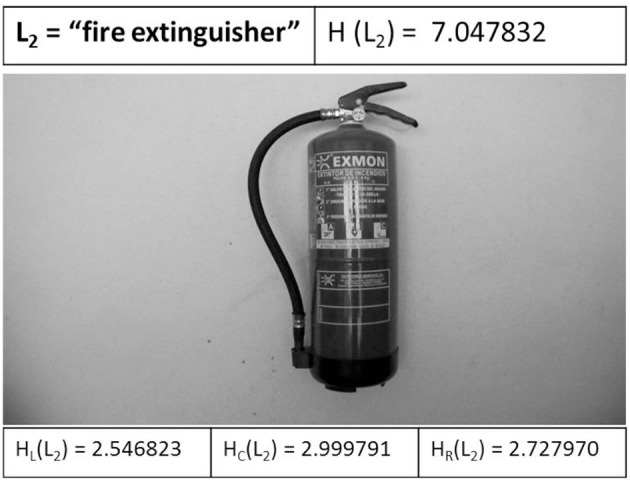
The next landmark L_2_ when is detected by the UAV.

Then, Figure 9 shows the next landmark L_2_ when is detected by the UAV. The entropic values are shown: the full entropy of image H(L_2_), the left entropy H_L_(L_2_), the center entropy H_C_(L_2_), and the right entropy H_R_(L_2_) of the image captured. H_C_(L_2_) is the higher entropy, therefore the UAV executes a maneuver go forward to the fire extinguisher.

Finally, the UAV reaches the target point or L_1_ landmark goal defined at the *k* = *19* iteration, and executes the specified maneuver of this arc: go forward to the goal, showing the exit door to an emergency situation.

**Figure 10:**
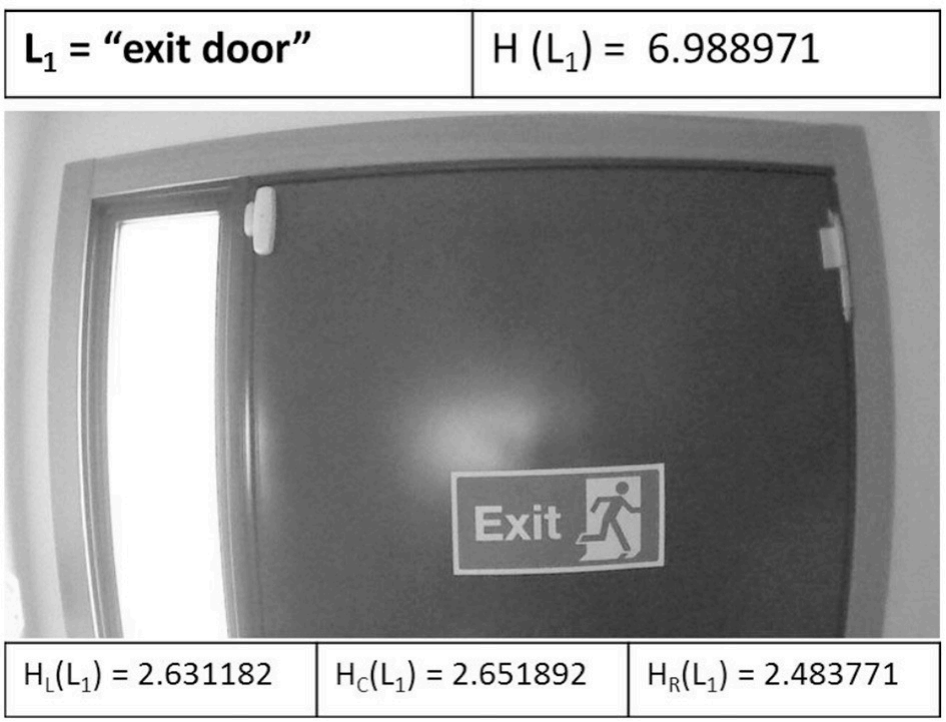
The target landmark L_1_ when is detected by the UAV.

Figure 10 shows the target landmark L_1_ when is detected by the UAV. The entropic values are shown: the full entropy of image H(L_1_), the left entropy H_L_(L_1_), the center entropy H_C_(L_1_), and the right entropy H_R_(L_1_) of the image captured. H_C_(L_1_) is the higher entropy, therefore the UAV executes a maneuver go forward to the exit door.

**Figure 11:**
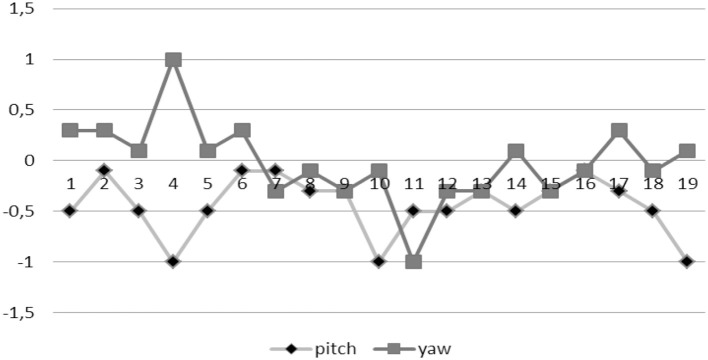
The control signals generated (*pitch* and *yaw*) in each *k* iteration.

During the experimentation, the UAV has used the *pitch* actuator (forward/back), the *gaz* actuator (up/down), and *yaw* actuator (rotation on its axis z). The values of the control signals u_*t*_ {pitch, yaw} that has been generated in each *k* iteration executed during the experiments, are shown in Figure 11. The control signals are generated (*pitch* and *yaw*) at each *k* iteration during the approximation maneuver of the UAV, from start point until to reach the target point (exit door). The landmarks L_3_, L_2_, and L_1_ are detected by the UAV at iteration *k* = *4, k* = *11*, and *k* = *19* respectively.

The entropy-based controller generates the values of the control signals through image entropy maximization (entropic vision), performing a maneuver for guide the robot to the higher entropy state in each iteration. When the left zone of image has the higher entropy (H_L_) the robot performs a turn to the left (*yaw* = [–1.0]), else if the right zone of image has the higher entropy (H_R_) the robot performs a turn to the right (*yaw* = [0.1]) in this case. If the higher entropy is centered on the image (H_C_), the robot goes forward (*pitch* = [–1.0]). The range of values for *yaw* and *pitch* are defined by the AR.Drone SDK (Piskorski et al., 2012).

The dual controller has generated the values of control signals adaptively through interaction of the robot with its environment. Experimentally, for the calculation of the combined signal u_t_, it has been established the following weight values: w_fb_ = 0.7 and w_ff_ = 0.3, for the feedback and feedforward controllers respectively (Maravall et al., 2015b).

From the experimental results obtained in our laboratory it is concluded that the UAV is able to successfully perform in real time the fundamental skills of the visual bug algorithm, guiding the robot toward a goal landmark (in this case exit door) using self-semantic location in each landmark defined in the visual topological map.

## Conclusions and future work

A hybrid algorithm for the self-semantic location and autonomous navigation of a robot based on entropic vision and the visual bug algorithm has been presented and tested in a scenario corresponding to a hypothetical emergency situation. The proposed algorithm uses a visual topological map to autonomously navigate in the environment. The nodes in the topological map determine a leave-point or a landmark for the self-location of the robot, in which the robot musts re-oriented its navigation in order to reach the goal. Unlike the classic bug algorithms, our algorithm does not require any knowledge about the robot's coordinates in the environment since the robot uses its own self-location method during the navigation to know its position in each iteration.

Based on the experimental results, it is concluded that this hybrid algorithm is highly robust when the robot is around an unknown location. The robustness is provided by the concept of entropic vision and the search of zones with high entropy. It is empirically confirmed the direct and positive correlation between the entropy of an image and the probability of the image of containing several objects inside. The performance when the robot encounters an obstacle during its navigation is acceptable, using the maximization of visual entropy as strategy. In addition, both techniques (the visual bug algorithm and the visual topological maps) together are able to increase the overall solution performance, reducing the number of iterations along the time for reach the goal landmark defined previously.

Future work is planned toward implementation of this hybrid algorithm on other situations in the real world, which an engineering process as self-semantic location of robots is needed: security in building, surveillance of frontiers or critical infrastructure control. We plan to develop further research work concerning the use of the UAV's onboard cameras for vision-based quality inspection and defects detection, taking profit of our experience in vision-based quality inspection and defects detection in the manufacturing industry, where we have introduced the novel concept of the histogram of connected elements as a generalization of the conventional gray level images histogram.

## Author contributions

The Authors DM, JdL, and JPF have been working about the paper titled: “Navigation and self-semantic location of drones in indoor environments by combining the visual bug algorithm and entropy-based vision” in the following tasks: definition of abstract, introduction, the dual FF/FB architecture, entropic-vision, and experimental works with drones.

### Conflict of interest statement

The authors declare that the research was conducted in the absence of any commercial or financial relationships that could be construed as a potential conflict of interest.
